# On the Use of Mustard in the Convulsions of Children

**Published:** 1844-07

**Authors:** Charles S. Tripler

**Affiliations:** Surgeon U. S. Army


*On the use of Mustard in the Convulsions of Children.* By Charles S. Tripler, M.D., Surgeon U. S. Army.—I had an obstinate case last summer, from teething, in which everything was tried, that I, or others, could think of, without success. As neither antimony, sulphate of zinc, sulphate of copper, ipecacuanha, nor any other emetic usually given, seemed to make any impression on the stomach, I thought I would try mustard, with a view to its emetic effects. In a few minutes it arrested a fearful attack of convulsions, that had lasted five hours; and that too, *without vomiting* the patient, for some time afterward.

A short time since I had three more cases in the course of a fortnight; with the first I tried the usual remedies, and I also made an ineffectual attempt at venesection (leeches could not be procured). The case still resisted, when the use of mustard again occurred to me. I gave it, and the patient was relieved in five minutes. With the other cases I used the mustard *at once*, and successfully. Its efficacy seems to have no relation to its emetic properties; for its sensible effect, in these cases, is as frequently to purge as to vomit. From my experience of the remedy, I do not hesitate to recommend its employment in these troublesome cases, in preference to any other internal remedy with which I am acquainted.—*Ibid.*

